# Identification of QTL with effects on intramuscular fat content and fatty acid composition in a Duroc × Large White cross

**DOI:** 10.1186/1471-2156-8-55

**Published:** 2007-08-16

**Authors:** Marie-Pierre Sanchez, Nathalie Iannuccelli, Benjamin Basso, Jean-Pierre Bidanel, Yvon Billon, Gilles Gandemer, Hélène Gilbert, Catherine Larzul, Christian Legault, Juliette Riquet, Denis Milan, Pascale Le Roy

**Affiliations:** 1 INRA, UR337 Station de génétique quantitative et appliquée, F-78350 Jouy-en-Josas, France; 2 INRA, UR444 Laboratoire de génétique cellulaire, F-31320 Castanet-Tolosan, France; 3 INRA, UE967 Génétique expérimentale en productions animales, F-17700 Surgères, France; 4 INRA, UAR2 Services déconcentrés d'appui à la recherche – Poitou-Charentes, F-17700 Surgères, France; 5 INRA-Agrocampus Rennes, UMR598 Génétique animale, F-35042 Rennes, France

## Abstract

**Background:**

Improving pork quality can be done by increasing intramuscular fat (IMF) content. This trait is influenced by quantitative trait loci (QTL) sought out in different pig populations. Considering the high IMF content observed in the Duroc pig, it was appealing to determine whether favourable alleles at a major gene or QTL could be found. The detection was performed in an experimental F2 Duroc × Large White population first by segregation analysis, then by QTL mapping using additional molecular information.

**Results:**

Segregation analysis provided evidence for a major gene, with a recessive Duroc allele increasing IMF by 1.8% in Duroc homozygous pigs. However, results depended on whether data were normalised or not. After Box-Cox transformation, likelihood ratio was indeed 12 times lower and no longer significant. The QTL detection results were partly consistent with the segregation analysis. Three QTL significant at the chromosome wide level were evidenced. Two QTL, located on chromosomes 13 and 15, showed a high IMF Duroc recessive allele with an overall effect slightly lower than that expected from segregation analysis (+0.4 g/100 g muscle). The third QTL was located on chromosome 1, with a dominant Large White allele inducing high IMF content (+0.5 g/100 g muscle). Additional QTL were detected for muscular fatty acid composition.

**Conclusion:**

The study presented results from two complementary approaches, a segregation analysis and a QTL detection, to seek out genes involved in the higher IMF content observed in the Duroc population. Discrepancies between both methods might be partially explained by the existence of at least two QTL with similar characteristics located on two different chromosomes for which different boars were heterozygous. The favourable and dominant allele detected in the Large White population was unexpected. Obviously, in both populations, the favourable alleles inducing high IMF content were not fixed and improving IMF by fixing favourable alleles using markers can then be applied both in Duroc and LW populations. With QTL affecting fatty acid composition, combining an increase of IMF content enhancing monounsaturated fatty acid percentage would be of great interest.

## Background

In the pig, intramuscular fat content (IMF) is considered to play a key role in organoleptic meat quality[1]. The increase of IMF is associated with an improvement in consumer perception of texture and taste [2]. Thus, in Large White and Landrace breeds, increasing IMF content, at least in the *Longissimus dorsi *muscle, is considered as highly desirable. Additionally, not only the amount of IMF has to be considered but also fatty acid composition, which is known to affect human health and also technological quality of fresh meat and sensory quality of pig meat products.

The existence of a gene with a great effect on IMF content was postulated by segregation analysis [3] in a Chinese European population. The gene, named *MI*, has a recessive allele (*imf*) that increases IMF content and originates from the Chinese Meishan breed. Duroc pigs, as well as Meishan pigs, are known for their high IMF content [4]. Moreover, in contrast to the Chinese pigs, Duroc animals have good performances of growth and body composition [5]. A previous analysis indicated the presence of a major gene affecting IMF content in Duroc population [6].

More recently, studies involving Duroc pigs have demonstrated the existence of a quantitative trait locus (QTL) affecting IMF located on chromosome 6 [7-9] but without mentioning a significant dominant/recessive effect. Other locations have also been reported for QTL affecting IMF content in different breeds [10-12].

The objective of the present work was to test the presence of a major gene affecting IMF content in a Duroc population using segregation analysis. Furthermore, molecular analysis has been performed on the same experimental animals in order to locate the putative gene on the pig genome and map QTL affecting fatty acid composition. For that purpose, an F2 resource population was created from Large White and Duroc pigs and measured for IMF characteristics.

## Results

### Segregation analysis

Segregation analysis, either with or without transformation of the data, revealed the presence of a recessive allele with a major effect on IMF content (Table 1). The mean difference between homozygous was estimated to be 1.5%, i.e. 2.1 phenotypic standard deviations. It should be noted that correction for skewness strongly decreased the likelihood ratio. When data were transformed, likelihood ratio was indeed 12 times lower and not significant anymore. The difference between homozygous dropped to 0.86% (1.2 phenotypic standard deviations). Estimated genotype frequencies revealed that only half the F1 breeding animals were heterozygous.

**Table 1:** Results from the segregation analysis

Parameters	μ_imf/imf_	μ_imf/IMF_	μ_IMF/IMF_	σ_e_	σ_g_	P_imf/imf_	P_imf/IMF_	*LR*	P
Without Box-Cox transformation	0.40	-1.10	-1.10	0.54	0.18	0.41	0.59	36.9	< 1.10^-6^
With Box-Cox transformation	1.56	0.70	0.70	0.53	0.20	0.39	0.61	3.1	0.53

μ_imf/imf_, μ_imf/IMF _and μ_IMF/IMF _= means of *imf/imf*, *imf/IMF *and *IMF/IMF *genotypes in g/100 g muscle, respectively, *imf *being the Duroc favourable allele; σ_e _= residual standard deviation; σ_g _= genetic standard deviation; *Pimf/imf *and *Pimf/IMF *= genotype frequencies of *AA *and *AB*, respectively; *LR *= likelihood ratio; *P *= level of significance.

### Detection of QTL

QTL analyses showed 3 chromosome-wide QTL affecting IMF content on chromosomes 1, 13 and 15 (Table 2). On SSC1, the allele responsible for an increased IMF content was dominant and from Large White origin. For the other two QTL on chromosomes 13 and 15, alleles responsible for an increased IMF were recessive and from Duroc origin. The favourable effect was of the same range for the three QTL.

**Table 2:** Results from the detection of QTLs with an effect on IMF

SSC	Maximum likelihood ratio	Location (cM)	Genotypic means (%)			Heterozygous boars (likelihood > 3.8, Khi2 with 1 DF)
			Du/Du	Du/LW	LW/LW	
1	16.4 (++)	16	-0.34	0.15	0.19	Boar 4
13	10.6 (+)	52	0.27	-0.09	-0.18	Boar 5
15	11.9 (+)	50	0.29	-0.12	-0.17	Boar 1, Boar 4

Maximum likelihood ratio significant at the 5% (+) or 1% (++) at the chromosome-wide level

Table 3 shows the location of significant QTL (at least at the chromosome-wide level) and their genetic effects on fatty acid composition. Only 4 QTL out of 47 were significant (P < 1%) at the genome-wide level, 3 located at similar positions on chromosome 14. The effect of alleles appeared to be additive. The Duroc/Duroc homozygous pigs had a lower percentage of C16:1, a higher percentage of C18:0, a higher percentage of saturated fatty acids and a higher chain length coefficient. The presence of pleiotropic QTL remains to be tested. QTL (p < 0.1% at the chromosome-wide level) were also found on chromosomes 7, 9, 10, and 15.

**Table 3:** Results of detection of QTLs affecting fatty acids composition

			Position	LR^3^	Genotypic mean (%)		
				
SSC	Trait^1^	P^2^	(cM)		Du/Du	Du/LW	LW/LW
1	C18:1	+	124	11.2	-0.79	0.36	0.43
1	C18:2 n-6	++	29	16.0	0.82	0.02	-0.84
1	MUFA	+	122	13.0	-0.93	0.38	0.55
1	P:S	+	29	12.4	3.90	-0.30	-3.60
2	C22:5 n-6	+	122	12.4	-0.03	-0.02	0.05
4	C22:5 n-6	+	117	12.9	-0.01	-0.03	0.04
5	MUFA	+	118	11.0	-0.49	-0.27	0.76
7	C18:1	+	66	13.9	0.43	0.51	-0.94
7	C18:2 n-6	+	60	15.0	-0.54	-0.37	0.91
7	C22:4 n-6	++	48	17.5	-0.04	-0.01	0.05
**7**	**C22:5**	*	**48**	**18.5**	**-0.05**	**0.00**	**0.05**
7	CLC	+	46	12.5	-0.02	-0.07	0.09
8	C16:0	+	66	14.1	-0.27	0.42	-0.14
9	C14:0	+	27	11.1	0.07	-0.05	-0.02
9	C18:0	+	14	10.8	-0.31	0.09	0.22
**9**	**C20:3**	*	**88**	**18.3**	**0.01**	**0.03**	**-0.04**
10	C16:1	+	106	12.7	0.12	0.03	-0.15
10	C18:1	+	103	13.4	0.62	0.34	-0.96
10	C18:2 n-6	+	108	11.1	-0.43	-0.40	0.83
**10**	**C20:5**	*	**27**	**17.4**	**0.01**	**-0.04**	**0.03**
10	C22:5 n-6	++	42	15.3	-0.01	-0.02	0.04
**10**	**MUFA**	*	**41**	**15.9**	**0.02**	**0.71**	**-0.73**
10	UC	+	42	11.6	0.00	-0.02	0.02
11	C17:1	+	92	12.4	-0.01	-0.09	0.11
11	C22:6 n-3	++	51	15.2	0.03	-0.01	-0.02
11	PUFA	+	89	13.6	1.41	-0.62	-0.80
11	P:S	+	63	13.2	4.73	-2.11	-2.62
11	UC	+	65	11.6	0.04	-0.02	-0.01
12	C16:1	+	40	11.6	-0.12	0.05	0.07
13	C18:1	+	45	12.2	0.81	-0.45	-0.36
13	C22:5 n-6	+	102	12.7	-0.02	-0.03	0.05
13	MUFA	+	45	14.6	0.96	-0.62	-0.34
14	C16:0	+	22	14.6	-0.07	0.33	-0.26
**14**	**C16:1**	**	**67**	**22.0**	**-0.19**	**0.04**	**0.15**
**14**	**C18:0**	***	**67**	**32.9**	**0.51**	**-0.09**	**-0.43**
14	C18:2 n-6	+	22	13.2	0.42	-0.78	0.36
14	C20:3 n-3	++	21	14.9	0.02	-0.03	0.01
14	C22:5 n-6	+	32	22.2	0.02	-0.03	0.01
**14**	**SFA**	**	**45**	23.7	0.62	0.11	-0.73
14	MUFA	+	67	12.3	-1.02	0.36	0.65
14	PUFA	+	23	12.6	0.73	-1.19	0.46
14	P:S	++	23	15.2	1.75	-4.06	2.30
14	UC	+	26	12.6	0.02	-0.03	0.01
**14**	**CLC**	**	**65**	**23.4**	**0.13**	**-0.04**	**-0.09**
**15**	**C14:0**	*	**44**	**18.4**	**0.08**	**-0.10**	**0.03**
16	C16:0	+	33	12.7	-0.13	-0.26	0.39
18	C20:5 n-3	+	7	11.9	-0.01	0.03	-0.01

^1^Percentages of saturated (SFA), monounsaturated (MUFA) and polyunsaturated (PUFA) fatty acids, unsaturation coefficient (UC), chain length coefficient (CLC), ratio of polyunsaturated fatty acids to saturated fatty acids (P:S); ^2^Maximum likelihood ratio significant at the 5% (+) or 1% (++) at the chromosome-wide level and in bold 5% (*), 1% (**) or 0.1% (***) at the genome wide level; ^3^LR: Maximum likelihood ratio

## Discussion

### Segregation analysis

The segregation analysis results illustrated the lack of robustness of segregation analysis in the presence of a skewed distribution. However, the decreased power of the analysis after the Box-Cox transformation has already been demonstrated [13]. The recessive allele, detected in the Duroc breed, had the same characteristics as the one detected in Meishan pigs [3]. The difference between homozygous pigs, obtained from non-transformed data, was indeed 2.1%, i.e. close to the value obtained here (1.5%).

### Detection of QTL

The detection of 3 QTL with effects of the same magnitude (difference between homozygous = 0.49, 0.43 and 0.44 phenotypic standard deviation for SSC1, SSC13 and SSC15, respectively) was unexpected. Moreover, one of the QTL was found to have a Large White favourable and dominant allele. The segregation analysis suggested the existence of only one large QTL with a favourable and recessive Duroc allele. In the segregation analysis, only 3 out of the 6 sires were probably heterozygous for the putative major gene. From these 3, 2 were also found heterozygous in the QTL detection. Boar 4 was not found heterozygous in the segregation analysis but was found to be heterozygous for both loci on SSC1 and SSC15 which showed opposite effects depending on the breed origin. Thus, it seemed that the major gene detected with the segregation analysis corresponded to the 2 QTL detected on chromosomes 13 and 15. The characteristics estimated from the QTL analysis were consistent with the segregation analysis even though their individual effects were weaker than the effect estimated previously. In a similar way, Janss et al (1997)  [3] did not locate one single QTL affecting IMF content in their crossbreeding experiment. If they evidenced one major gene by segregation analysis, afterwards they located several small QTL with reduced individual effects.

The chromosome 1 QTL could not have been detected by segregation analysis because only one boar could be considered as heterozygous for this QTL, which was also heterozygous for the QTL on SSC15.

These QTL locations did not correspond to QTL locations generally reported in the literature for related traits. QTL mapping involving the Meishan breed located QTL for intramuscular fat content on SSC2, SSC4, SSC6, SSC7 and SSCX [10,12,14]. Two studies involving the Duroc breed [7,8] did not evidence a Duroc recessive allele because they involved backcross design. A QTL was located on chromosome 13 in an F2 Meishan × Duroc population [14], at position 117 cM with a favourable recessive Duroc allele (additive effect = 0.95%). Stearns et al (2005) [15] evidenced a QTL on chromosome 13 with an additive effect at 88 cM, the allele inherited from the Duroc line being associated to higher lipid content. Rohrer et al (2005) [16] also mentioned a suggestive QTL (without any further details) for intramuscular fat content on chromosome 13 in a Duroc × Landrace F2 population at 53 cM. They reported, at the same location, a dominant QTL for moisture content, with the Duroc allele associated to lower moisture content. Knowing the high negative correlation between moisture and lipid content, it should be assumed that for lipid content, the QTL would also be dominant with a Duroc allele associated with a higher intramuscular fat content. Additionally to the present study, extensive muscular characteristics were measured on a subset of F2 animals with extreme IMF content [17]. The two groups essentially differed by the number of muscular adipocytes. Previously, it was observed that Duroc and Landrace pigs might show clear differences in peroxisome proliferator-activated receptor gamma (*PPARγ*) expression [18]. Furthermore, the *PPARγ *gene maps to porcine chromosome 13 [19]. Unfortunately, Grindflek et al (2004) [20] did not evidence any association between *PPARγ *polymorphism and IMF content, but the studied populations would fail to fit an association with a recessive Duroc allele. Kim et al (2004) [21] studying Landrace, Large White, Duroc, Berkshire and synthetic Duroc × Large White populations failed to find any polymorphism in the *PPARγ *gene. However, these authors suggested an association between marbling score and polymorphism in the *GHRL *gene, mapped close to *PPARγ *on chromosome 13, in a Berkshire population.

Considering the two QTL located on SSC1 and SSC15, some studies with different breeds showed similar results. A partially dominant Yorkshire allele with an increasing effect on IMF content has been detected in an F2 Berkshire × Yorkshire population. It was located on chromosome 1, at 16 cM [22]. As shown by [16] on SSC4 in a Landrace × Duroc crossbreeding, recessive allele associated with high intramuscular fat content could also be found in Landrace. Two QTL affecting IMF content were located on chromosome 15, at 42 and 57 cM, in an F2 wild boar × Large White cross [23]. For that QTL, the dominance effect was very low and as expected the Large White allele increased IMF content.

The most significant QTL were linked to fatty acid composition. Location of several QTL was to be expected considering the high heritability values of fatty acid traits [24]. Previous studies reported QTL on fatty acid composition measured in the backfat [25,26] but the present study is the first one to present QTL on fatty acid composition measured in pig meat. In [25], QTL were found on chromosomes 4, 6, 8, 10 and 12. In [26], they found QTL on chromosomes 1, 2, 3, 4, 5, 6, 9, 14, 15, 16, 17 and X. Considering the low genetic correlations between muscle and backfat characteristics [24], QTL were expected to be different. As mentioned by [27] in pigs, fatty acid composition is altered by food composition. The polyunsaturated linoleic and α-linolenic fatty acids cannot be synthesised *in situ*, thus tissue concentrations respond rapidly to dietary changes. In contrast, saturated and monounsaturated fatty acids are *de novo *synthesised, hence their concentrations are less readily influenced by diet. Fatty acid composition is also influenced by genetic background. Duroc pigs have a higher concentration of saturated and monounsaturated fatty acids than Landrace pigs [28]. QTL located on SSC14 showed an increase of saturated fatty acids associated with a Duroc allele. Interestingly, a suggestive QTL located on the same chromosome affecting monounsaturated fatty acid showed a Duroc allele associated to a lower concentration of monounsaturated fatty acids. A model fitting one pleiotropic or two QTL should be performed to determine if this is the same QTL. On SSC13, close to one of the IMF QTL, the suggestive QTL affecting the concentration of monounsaturated fatty acids showed a Duroc allele associated to a favourable but recessive effect. The existence of one or two different QTL has to be tested. Location of the QTL detected on SSC12 should be considered with cautious because no genotype information was available at the extreme of the chromosome.

Influencing fatty acid composition could be of interest considering pork nutritional quality, especially those influencing mono- and polyunsaturated fatty acids. It is known that a higher percentage of polyunsaturated fatty acids leads to a meat of inferior quality. This might be related to softness and oxidation phenomena, producing off-flavours and rancidity in meat. From a dietary point of view, a lower percentage of saturated fatty acids is considered as beneficial, because the main fatty acids behind the cholesterol elevating effect are C14:0 and C16:0. Combining an increase of IMF content enhancing monounsaturated fatty acid percentage would be of great interest.

## Conclusion

The present study indicated a complementary approach of a segregation analysis and a QTL detection to seek out genes involved in the higher IMF content observed in the Duroc population. Discrepancy between both methods might be partially explained by the existence of at least two QTL with the same properties located on two different chromosomes for which different boars were heterozygous. The favourable and dominant allele detected in the Large White population was completely unexpected. In both populations, the favourable alleles inducing high IMF content were not fixed. Improving IMF by fixing favourable alleles using markers can then be applied both in Duroc and LW populations. However, a more accurate location should be performed before. Additionally, this study provided for the first time evidence for QTL on muscle fatty acid composition, with leads to modify both IMF content and monounsaturated fatty acid percentage.

## Methods

### Animals

An F2 cross between Duroc and Large White pigs was performed at the INRA experimental farm of Le Magneraud (Charente-Maritime, France). A total of 204 Duroc pigs (117 boars and 87 sows) issued from the nucleus herd of Selpa (Alliers, France) was measured for IMF content. Among these Duroc animals, 8 boars with high IMF content values (from 3.3 to 5.8%) were selected and mated to 37 Large White sows (F0 generation). Among F1 animals, 10 sires and 32 dams were randomly selected to produce the next generation. Each F1 dam produced 3 litters with the same boar and a total of 775 F2 pigs were generated. All the F2 piglets were weaned at 28 days of age and placed in post weaning collective pens until 10 weeks of age.

### Intramuscular fat measurements

IMF content was measured in Duroc, F1 and F2 animals. At a live weight of 70 kg, a muscle sample was taken by biopsy from the *longissimus dorsi *muscle at the level of the last rib. Lipids were extracted from 1 g samples [29] and weighed. IMF content is expressed in g/100 g muscle. All analyses were done in duplicate. Mean results from the two samples analysed were used for statistical analyses (Table 4).

**Table 4:** Means (in %) and Standard deviation (SD) of data

Variable	Label	Mean	SD
c14:0	Myristic acid	0.68	0.54
c16:0	Palmitic acid	23.4	1.3
c16:1	Palmitoleic acid	2.85	0.55
c17:1	Heptadecaenoic acid	0.429	0.820
c18:0	Stearic acid	11.8	1.3
c18:1	Oleic acid	38.8	3.2
c18:2 n-6	Linoleic acid	15.7	2.6
c18:3 n-3	Linolenic acid	0.717	0.303
c20:0	Arachidic acid	0.154	0.091
c20:1	Eicosaenoic acid	0.566	0.145
c20:2	Eicosadienoic acid	0.517	0.117
c20:3 n-6	Di-homo γ linolenic	0.337	0.136
c20:4 n-6	Arachidonic acid	2.79	0.80
c20:5 n-3	Eicosapentaenoic acid (EPA)	0.171	0.107
c22:4 n-6	Docosatetraenoic acid	0.420	0.142
c22:5 n-6	Docosapentaenoic acid	0.455	0.147
c22:6 n-3	Docosahexaneoic acid (DHA)	0.186	0.100
SFA	% saturated fatty acids	36.1	2.0
MUFA	% monounsaturated fatty acids	42.6	3.5
PUFA	% polyunsaturated fatty acids	21.6	3.8
P:S	Polyunsaturated to saturated ratio	0.604	0.122
INSAT	Unsaturation coefficient	1.50	0.10
CLC	Chain length coefficient	17.6	0.06
IMF	Intramuscular Fat	2.03	0.67

The fatty acid composition of lipid fractions was determined by gas-liquid chromatography of methyl esters [30]. Fatty acid composition was expressed in % total fatty acids. The following parameters were calculated from fatty acid composition:

- the sum of saturated, monounsaturated and polyunsaturated fatty acids;

- the ratio of polyunsaturated fatty acids to saturated fatty acids;

- the unsaturation coefficient defined as the average number of double bounds of unsaturated fatty acids;

- the chain length coefficient calculated using the formula Σp_i_c_i_/100 where p_i _and c_i _are respectively the percentage and the number of carbon atoms of each fatty acid i;

- the ratio of n-6 fatty acids to n-3 fatty acids.

### Molecular analyses

The 6 F1 boar families with the largest number of offspring were selected for molecular analyses. F1 sires were genotyped for 157 molecular markers. Among these markers, a set of 91 informative markers covering the porcine autosomes (from 3 to 9 markers per chromosome; Table 5) was selected. Some chromosomal ends (4, 5, 6, 12, 15) were not covered by genotype markers. Thirty-one F0 animals, 27 F1 animals (6 boars and 21 sows) and 456 F2 pigs were genotyped for this set of markers using DNA extracted from blood samples.

**Table 5:** Location of markers

	Chr	cM		Chr	cM		Chr	cM
SW552	1	9	S0383	7	0	SW1307	12	40
S0008	1	44	S0025	7	24	SW874	12	63
S0396	1	60	SW1354	7	42	S0090	12	80
S0155	1	95	LRA1	7	75	SW2180	12	105
SW1828	1	120	S0102	7	90	SWR1941	13	14
SW1301	1	140	SW352	7	108	S0222	13	45
SWC9	2	1.1	SW632	7	125	SW225	13	70
SW2623	2	10	S0101	7	155	SW38	13	102
SW240	2	41	SW764	7	175	SW857	14	8
S0226	2	79	SW2410	8	1	S0058	14	32
S0368	2	105	SW905	8	20	S0007	14	60
S0036	2	132	SWR1101	8	37	SW55	14	80
SW72	3	17	SW1843	8	53	SW2515	14	108
SW487	3	42	SW1551	8	106	SW1111	15	27
SW102	3	65	S0178	8	127	S0088	15	53
S0372	3	95	SW983	9	1	SW936	15	79.4
S0397	3	110	SW911	9	32	S0111	16	1
SW2547	4	30	SW940	9	57	SW2411	16	17
SW839	4	62.1	SW1677	9	76	S0026	16	47
S0214	4	80	SW2093	9	100	SW1897	16	86
SW445	4	105	SW2116	9	126	SW24	17	23
S0097	4	120	SWR136	10	8	SW2441	17	41
SW1482	5	39	SW2491	10	42	SW1920	17	56
SW2425	5	72	S0070	10	62	S0359	17	75
SW1987	5	102	SW951	10	95	SW2540	18	2
IGF1	5	118	SWR67	10	121	SW1984	18	30
SW378	5	133	S0392	11	3	S0120	18	45
SW1353	6	29	SW1632	11	16	SW980	X	11
S0087	6	62	S0382	11	52	SW1903	X	32
SW122	6	84	SW1377	11	76	SW2456	X	58
S0228	6	105	SW1135	11	100	SW1994	X	75
S0121	6	116				SW1943	X	87
SW2419	6	161				S0218	X	115

### Statistical analyses

Prior to segregation (without marker information) or QTL detection (with marker information) analyses, intramuscular fat content and fatty acid composition traits were adjusted for the effects of contemporary group, sex and age at biopsy with the GLM procedure of SAS [31].

First, a segregation analysis was applied to adjusted data [32]. A description of the model used and the details on calculations are given in Sanchez et al. [33]. Briefly, this method was based on the comparison of the likelihood of the data under a mixed model (*H1*: a major gene + polygenes) and under a polygenic model (*H0*). We assumed that the data originated from independent sire families. The major gene was modelled as an autosomal biallelic (*A *and *B*) locus with Mendelian transmission probabilities. Three genotypes could thus be encountered: *AA*, *AB *and *BB*. Under *H1*, the model depended on 7 parameters: *μ*_*AA*_, *μ*_*AB *_and *μ*_*BB *_(genotype means), *σ*_*g *_and *σ*_*e*_(genetic and residual standard deviations, respectively), *P*_*AA *_and *P*_*AB *_(genotype frequencies). *H0 *was a sub-hypothesis of *H1 *and was given by *μ*_*AA *_= *μ*_*AB *_= *μ*_*BB *_= *μ*_0_. Under *H0*, the model thus depended on 3 parameters (*μ*_0_, *σ*_*g *_and *σ*_*e*_). The test statistic was a likelihood ratio *l *= -2 ln (*M0*/*M1*) where *M1 *and *M0 *were the maximised likelihoods under *H1 *and *H0*, respectively. We supposed that the likelihood ratio was asymptotically distributed as a χ^2 ^with 4 degrees of freedom [34].

**Figure 1:**
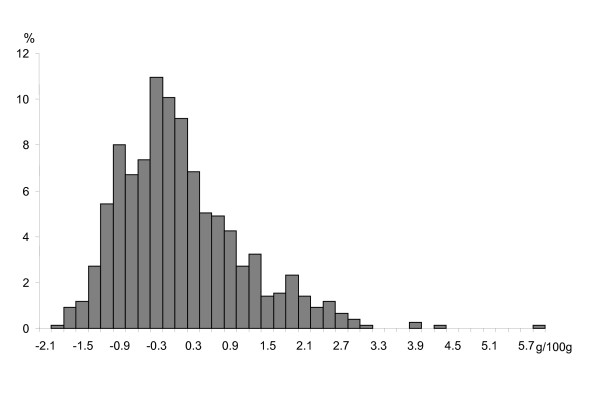
Distribution of adjusted intramuscular content in the F2 Duroc × Large White population.

The F2 distribution of adjusted IMF content appeared strongly skewed (Figure 1, 6). A skewed distribution is indeed expected in F2 when a major gene is segregating with non-additive effect. However, skewness may lead to a false inference of a major gene [13]. In order to resolve between skewness of the trait and segregation at a major locus, penetrance functions were written under *H0 *and *H1 *using a Box-Cox transformation [35]. The transformation parameters were estimated jointly with the other model parameters, under both *H0 *and *H1 *hypotheses. In order to test the effect of the Box-Cox transformation on segregation analysis results, segregation analysis was also performed without Box-Cox transformation.

Second, interval mapping analyses were performed to detect QTL using molecular information on adjusted data, with the QTLMAP software developed at INRA [36]. For successive locations, the hypothesis of one QTL (H1) in segregation at the given position was compared to the hypothesis of no QTL (H0) on the chromosome. The model assumed that alternative QTL alleles A and B were fixed in each founder population. Performance distributions were modelled within families, allowing heterogeneity of variance between sire families. Under the H1 hypothesis, a QTL with additive and dominance effects was fitted to the data, so that means of trait distributions depending on the QTL genotype were estimated: μAA, μAB, μBB. Likelihoods were maximised under each hypothesis, and a likelihood ratio test (L-ratio) was computed for each position tested. Significance thresholds were empirically determined at the chromosome level, by performing simulations under H0, using the assumption of a polygenic infinitesimal model [36]. A total of 5,000 simulations were achieved for each chromosome, and an approximate Bonferroni correction was applied to obtain genome-wide significance levels.

## Authors' contributions

MPS performed statistical analyses (segregation analysis, final QTL detection) and drafted the manuscript. BB performed the first draft of QTL location. YB supervised the performance testing, from animal production to biological sampling. CL helped to draft the manuscript and finalized the writing. HG advised the QTL detection analysis. NI genotyped F0 and F1 animals to identify informative markers, DM selected markers to screen the whole genome and supervised genotyping work and JR checked haplotypes of fat animals in QTL regions. GG, CLe and JPB participated in the design and the realisation of the experiment. PL had the scientific responsibility and coordinated the whole experiment.
